# Causal relationship between immune cells and risk of myocardial infarction: evidence from a Mendelian randomization study

**DOI:** 10.3389/fcvm.2024.1416112

**Published:** 2024-08-27

**Authors:** Wenjing Cao, Kui Wang, Jiawei Wang, Yuhua Chen, Hanxian Gong, Lei Xiao, Wei Pan

**Affiliations:** ^1^ Cardiology Department, Geriatrics Department, Foshan Women and Children Hospital, Foshan, Guangdong, China; ^2^ Department of Gastroenterology, The First People’s Hospital of Yunnan Province, The Affiliated Hospital of Kunming University of Science and Technology, Kunming, Yunnan, China; ^3^ Medical School, Kunming University of Science and Technology, Kunming, Yunnan, China; ^4^ Department of Critical Care Medicine, Jieyang Third People’s Hospital, Jieyang, China; ^5^ The First Clinical Medical College, Lanzhou University, Lanzhou, China

**Keywords:** causal inference, myocardial infarction, genome-wide association study, MR analysis, immunity

## Abstract

**Background:**

Atherosclerotic plaque rupture is a major cause of heart attack. Previous studies have shown that immune cells are involved in the development of atherosclerosis, but different immune cells play different roles. The aim of this study was to investigate the causal relationship between immunological traits and myocardial infarction (MI).

**Methods:**

To assess the causal association of immunological profiles with myocardial infarction based on publicly available genome-wide studies, we used a two-sample mendelian randomization (MR) approach with inverse variance weighted (IVW) as the main analytical method. Sensitivity analyses were used to assess heterogeneity and horizontal pleiotropy.

**Results:**

A two-sample MR analysis was conducted using IVW as the primary method. At a significance level of 0.001, we identified 47 immunophenotypes that have a significant causal relationship with MI. Seven of these were present in B cells, five in cDC, four in T cells at the maturation stage, six in monocytes, five in myeloid cells, 12 in TBNK cells, and eight in Treg cells. Sensitivity analyses were performed to confirm the robustness of the MR results.

**Conclusions:**

Our results provide strong evidence that multiple immune cells have a causal effect on the risk of myocardial infarction. This discovery provides a new avenue for the development of therapeutic treatments for myocardial infarction and a new target for drug development.

## 1 Introduction

Myocardial infarction (MI) occurs on the basis of atheromatous coronary artery lesions, where there is a dramatic reduction or interruption of coronary blood supply, leading to myocardial ischemia and hypoxia or even necrosis (1, 2). Cardiovascular disease is the leading cause of death in the United States, with a prevalence of myocardial infarction as high as 3.2 per cent among American adults (3). Globally, more than 8 million lives are threatened by AMI each year, resulting in a huge health and economic burden (4–6).

Recent studies have shown that the immune system plays a key role in myocardial infarction development and repair, determining the extent of myocardial damage and prognosis (7–9). Intense aseptic inflammation occurs in the infarcted area, which can be divided into an inflammatory phase, a proliferative phase and a mature phase (10, 11). Neutrophils, macrophages and immune cells such as B and T cells are attracted to the infarcted area by chemokines and produce pro-inflammatory or anti-inflammatory factors to remove and repair damaged tissue, affecting cardiac remodeling and healing (12–14). Macrophages are the most numerous immune cells in myocardial tissue (14, 15). Cardiac macrophages can be divided into two subpopulations depending on their origin (16). Macrophages of monocyte origin are predominantly associated with inflammatory responses and pathogenic properties, while resident macrophages of embryonic yolk sac origin exhibit cardioprotective functions. Early macrophages produce pro-inflammatory cytokines leading to tissue inflammation and over time can polarize into M2 macrophages that release anti-inflammatory cytokines to repair the healing process (14). Studies analyzing the role of different subsets of innate lymphoid cells (ILC) reveal the involvement of ILC1 in the progression of atherosclerosis (AS) and the anti-AS role of ILC2 (17). Rafael Blanco-Domínguez finds that CD69 expression on Tregs increases survival from coronary ligation in mice, and increased myocardial inflammation and deterioration of cardiac function after ischemia in CD69- mice (18). These findings suggest that different subpopulations of immune cells behave differently in the inflammatory response to myocardial infarction (8, 14, 17, 19, 20). It has been found that an overactive inflammatory response leads to excessive fibrosis appearing as heart failure with preserved ejection fraction. The absence of an effective inflammatory response leads to unstable scarring and ventricular rupture (13, 20). However, there are few studies on different immune cell subsets and myocardial infarction. These studies often suffer from the limitations of limited sample size, confounding factors and reverse causality.

Mendelian randomization is a statistical technique. Genetic variants, also known as single nucleotide polymorphisms (SNPs), are used as instrumental variables(IV). MR uses genetic variants as a proxy for exposure to assess whether exposure is associated with an outcome (21). It is well known that genetic variants are randomly assigned at conception. In this way, MR is similar to a randomized controlled trial (RCT), which avoids confounding factors and reverse causality. It follows that MR can provide a causal relationship between exposure and outcome (22–25). The role of different types of immune cells in the development and progression of atherosclerosis has been confirmed in previous observational studies. This study identifies additional immune cells associated with myocardial infarction and elucidates the causal relationship between these immune traits and myocardial infarction. The discovery of the immune cell traits associated with the occurrence of MI will help to develop novel therapeutic targets for therapeutic interventions in myocardial infarction (26).

## 2 Materials and methods

### 2.1 Study design

**Figure 1:**
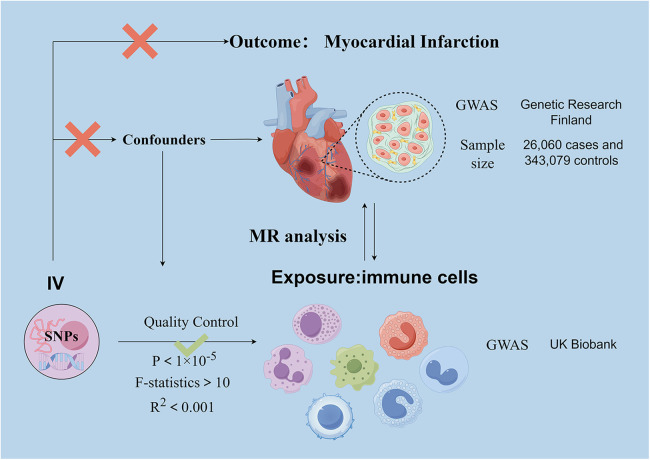
Schematic representation of the research process. Assumption 1, significantly associated with exposure, Assumption 2, not associated with outcome, Assumption 3, not associated with confounders. SNPs, single-nucleotide polymorphisms; MR, Mendelian randomization.

We analyzed the causal relationship between 731 immune cell traits and myocardial infarction based on two-sample Mendelian randomization (MR). MR studies use genetic variation that is associated with exposure as an instrumental variable (IV) to predict the causal relationship between exposure and outcome (27). The study obtained 47 genetic variants for circulating immune cell traits and their association with the risk of myocardial infarction (MI) from previously published genome-wide association studies (GWAS).The use of MR design reduces the impact of confounding and reverse causation, thereby improving the ability to make causal inferences about associations between exposure and outcome. The use of genetic variants as instrumental variables (IV) to study the effects of altered exposures is advantageous due to their random assignment at the time of conception, making them less susceptible to confounding by environmental factors or reverse causation. This study analyzed datasets from human individuals that are publicly available. The overall design is shown in Figure 1. As it is a secondary analysis of published data, ethical approval is not required.

### 2.2 Genome-wide association study (GWAS) data sources for MI

The genome-wide association study (GWAS) summary data for MI are sourced from the December 2023 released by Genetic Research Finland and can be accessed at https://finngen.gitbook.io/documentation. The FinnGen study has analyzed more than 500,000 biobank samples from Finland. The study correlates genetic variants with health data to understand pathogenesis and identify risk factors. A genome-wide association study (GWAS) was conducted on 118,870 European individuals, with 26,060 cases and 343,079 controls.

### 2.3 Immunity-wide GWAS data sources

Genetic variants associated with immune cells are derived from pooled data from the UK Biobank. This information is available for search on the website https://gwas.mrcieu.ac.uk/datasets/.GWAS statistical summaries for each immunological trait are publicly available from the GWAS catalogue under accession numbers ranging from GCST90001391 to GCST90002121. The GWAS data comprised four cellular parameters: median fluorescence intensity (MFI) representing surface antigen levels (*n* = 389), absolute cell count (AC) (*n* = 118), morphological parameters (MP) (*n* = 32), and relative cell count (RC) (*n* = 192). Of these, AC, MFI and RC features comprised B cells, CDC, T cell maturation stage, monocytes, myeloid cells, TBNK (T cells, B cells, natural killer cells) and Treg panels, whereas MP features included CDC and TBNK panels.

### 2.4 Selection of instrumental variables (IVs)

In causal inference, a valid instrumental variable (IV) must satisfy three key assumptions: (1) the genetic variant is directly related to the exposure (731 circulating immune cell traits); (2) there is no confounding influence between the genetic variant and the outcome; and (3) the genetic variant does not affect the outcome in ways other than through the exposure. Based on recent studies, a significance level of 1 × 10^−5^ was used for the selection of instrumental variables (IV) associated with each immunological trait. To ensure unbiased results, we applied a chain imbalance threshold of 5,000 kb and an *R*^2^ value of less than 0.001 to the chain imbalance distance. The strength of the association between the independent variables (IVs) and exposure was assessed using the F-statistic. IVs with an F-statistic greater than 10 were used to exclude effects due to weak instrumental variables (28).

### 2.5 Statistical analysis

A variety of MR analyses were conducted, such as MR Egger, weighted median, inverse variance weighted (IVW), simple mode, weighted mode, and MR-PRESSO methods. Each of these methods uses different statistical assumptions to make causal inferences (29). In MR analysis, the primary method for assessing causality is IVW (30). IVW assumes that all SNPs are independent of pleiotropy and depend on the variance of the outcome, and this method has a robust causality detection (31). Even if the results of the weighted median and MR-Egger methods were not significant, we considered them significant if the IVW results were significant (*p* < 0.05) and consistent with the trend of the other methods (32, 33). The odds ratio (OR) reflects the increase in risk factor levels per standard deviation (SD) (25). We used the Cochrane Q statistic to assess the variability of the instrumental variables, with *p*-value above 0.05 indicating no heterogeneity. To assess the presence of horizontal pleiotropy, we conducted MR Egger regression analysis. A *p*-value greater than 0.05 suggests the absence of horizontal pleiotropy (34). A “leave-one-out” analysis evaluate the impact of individual SNPs on the overall MR estimate. Furthermore, scatter plots were utilized to demonstrate that outliers did not impact the outcomes (35).

## 3 Results

### 3.1 An overview of IVs

According to recent studies, the genome-wide significance *p*-value for each immune cell trait in IVs was set at 5 × 10^−6^ (36–38). Following F-statistical validation, 985 SNPs were identified as IVs among 731 immune cell SNPs. The retained SNPs were found to be strong instruments, as indicated by F-statistics exceeding 10. These results are presented in Supplementary Table S1.

### 3.2 Exploration of the causal effect of immunophenotypes and MI

**Figure 2:**
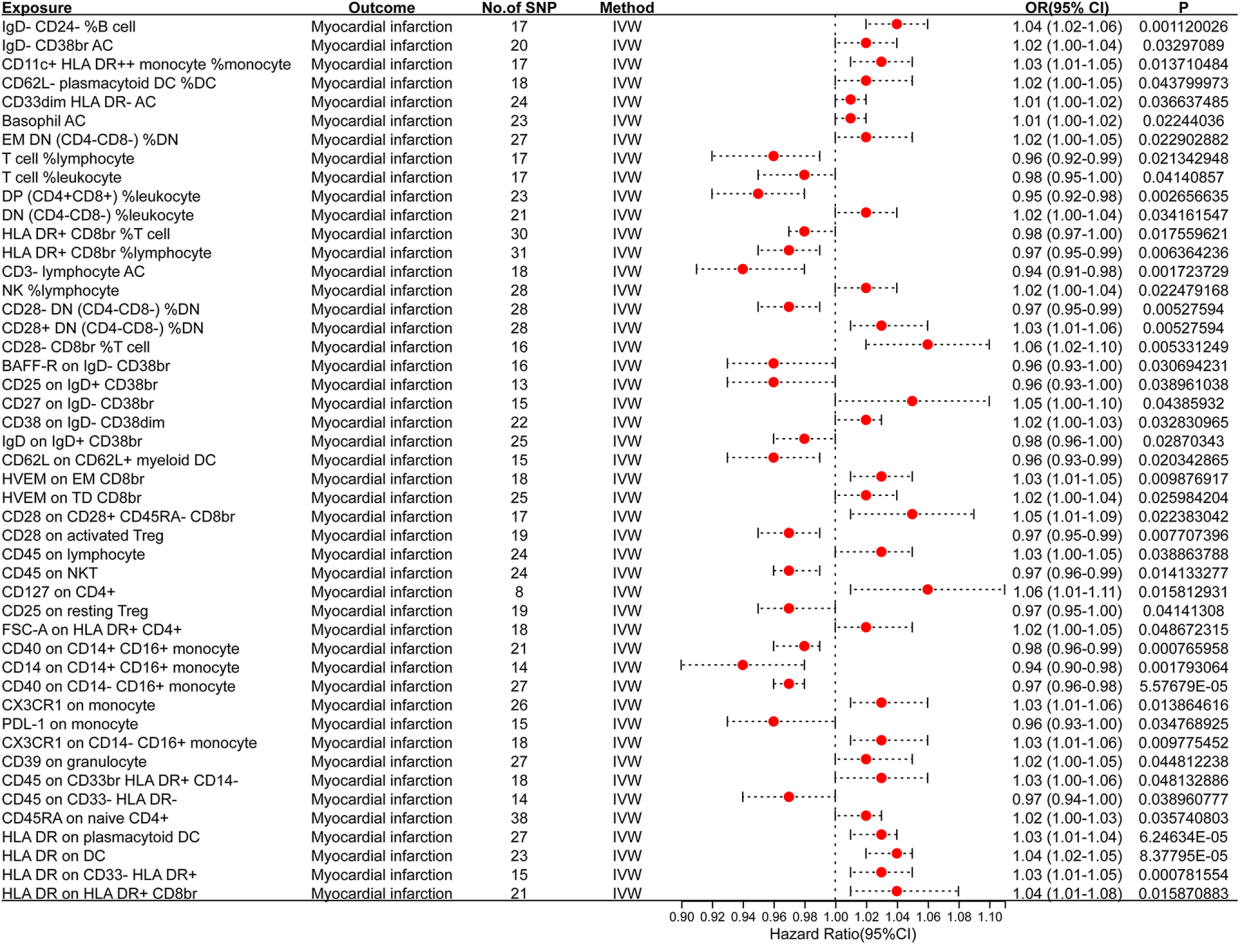
Forest plot of the MR analysis results.

To investigate the causal relationship between immunophenotype and MI, we conducted a two-sample MR analysis using IVW as the primary method. At a significance level of 0.001, we identified 47 immunophenotypes that have a significant causal relationship with MI. Of these, 7 were found in B cells, 5 in cDC, 4 in the maturation stages of T cells, 6 in monocytes, 5 in myeloid cells, 12 in TBNK cells, and 8 in Treg cells. Detailed findings are presented in Table 1 and Figure 2. We conducted MR-Egger intercept and MR-PRESSO global tests to eliminate SNPs with pleiotropy (MR-PRESSO test *P* < 0.05, MR-Egger regression *P* < 0.05). Following Cochran’s *Q* test, SNPs with heterogeneity (*P* < 0.05) were excluded, as detailed in Table 1. Furthermore, the leave-one-out analysis indicated that the exclusion of any of the SNPs did not affect the overall results. This suggests a stable causal relationship between immunophenotype and MI (Supplementary Figure S1**)**. In addition, the stability of causality is demonstrated by analysing species using scatterplots and funnel plots. (Supplementary Figure S2**)**.

**Table 1:** Summary of the GWAS included in this two sample Mendelian randomization study.

Panel	immune cells	Outcome	Nsnp	Methods	Beta	OR (95% CI)	*P* value	Heterogeneity		Horizontal pleiotrop	
								Cochran’s Q	*P* value	Egger intercept P	Global_P
B cell	IgD- CD24- %B cell	Myocardial infarction	17	Inverse variance weighted	0.037810369	1.04 (1.02–1.06)	0.001120026	18.00520728	0.323592124	0.291426349	0.398333333
B cell	IgD- CD38br AC	Myocardial infarction	20	Inverse variance weighted	0.022167148	1.02 (1.00–1.04)	0.03297089	27.14380966	0.101347707	0.233165063	0.151
B cell	BAFF-R on IgD- CD38br	Myocardial infarction	16	Inverse variance weighted	−0.037138215	0.96 (0.93–1.00)	0.030694231	13.21754948	0.585498791	0.105054123	0.592666667
B cell	CD25 on IgD + CD38br	Myocardial infarction	13	Inverse variance weighted	−0.039353046	0.96 (0.93–1.00)	0.038961038	15.95386801	0.193358141	0.103017285	0.161333333
B cell	CD27 on IgD- CD38br	Myocardial infarction	15	Inverse variance weighted	0.047099732	1.05 (1.00–1.10)	0.04385932	10.32121507	0.738342137	0.630810454	0.765333333
B cell	CD38 on IgD- CD38dim	Myocardial infarction	22	Inverse variance weighted	0.015234395	1.02 (1.00–1.03)	0.032830965	11.56475712	0.950629904	0.671168903	0.963666667
B cell	IgD on IgD + CD38br	Myocardial infarction	25	Inverse variance weighted	−0.023611417	0.98 (0.96–1.00)	0.02870343	23.97965348	0.462761319	0.575552103	0.525666667
cDC	CD11c + HLA DR++ monocyte %monocyte	Myocardial infarction	17	Inverse variance weighted	0.026107887	1.03 (1.01–1.05)	0.013710484	13.10249677	0.665249267	0.264441394	0.581666667
cDC	CD62l- plasmacytoid DC %DC	Myocardial infarction	18	Inverse variance weighted	0.022621383	1.02 (1.00–1.05)	0.043799973	22.50851263	0.165945162	0.212147372	0.175
cDC	CD62l on CD62l + myeloid DC	Myocardial infarction	15	Inverse variance weighted	−0.041700673	0.96 (0.93–0.99)	0.020342865	9.762419198	0.779338874	0.768404213	0.793
cDC	HLA DR on plasmacytoid DC	Myocardial infarction	27	Inverse variance weighted	0.028988572	1.03 (1.01–1.04)	6.24634E-05	21.26372906	0.728225703	0.696403886	0.585
cDC	HLA DR on DC	Myocardial infarction	23	Inverse variance weighted	0.034598717	1.04 (1.02–1.05)	8.37795E-05	29.45100187	0.13242949	0.307270679	0.123333333
Maturation stages of T cell	EM DN (CD4-CD8-) %DN	Myocardial infarction	27	Inverse variance weighted	0.023864011	1.02 (1.00–1.05)	0.022902882	30.26350364	0.256838399	0.704803743	0.261333333
Maturation stages of T cell	HVEM on EM CD8br	Myocardial infarction	18	Inverse variance weighted	0.026546793	1.03 (1.01–1.05)	0.009876917	15.48053223	0.560911502	0.931892539	0.618666667
Maturation stages of T cell	HVEM on TD CD8br	Myocardial infarction	25	Inverse variance weighted	0.02092931	1.02 (1.00–1.04)	0.025984204	34.69949821	0.072982692	0.639322374	0.074666667
Maturation stages of T cell	CD45RA on naive CD4+	Myocardial infarction	38	Inverse variance weighted	0.016066387	1.02 (1.00–1.03)	0.035740803	45.73094659	0.153684385	0.415481393	0.173333333
Monocyte	CD40 on CD14+ CD16 + monocyte	Myocardial infarction	21	Inverse variance weighted	−0.024301295	0.98 (0.96–0.99)	0.000765958	14.41762759	0.80870593	0.131828876	0.792666667
Monocyte	CD14 on CD14+ CD16 + monocyte	Myocardial infarction	14	Inverse variance weighted	−0.066148154	0.94 (0.90–0.98)	0.001793064	12.81345799	0.462321872	0.701184772	0.542333333
Monocyte	CD40 on CD14- CD16 + monocyte	Myocardial infarction	27	Inverse variance weighted	−0.029940204	0.97 (0.96–0.98)	5.57679E-05	29.39315625	0.293509109	0.512883771	0.356666667
Monocyte	CX3CR1 on monocyte	Myocardial infarction	26	Inverse variance weighted	0.032314234	1.03 (1.01–1.06)	0.013864616	35.88710325	0.073330422	0.732726987	0.086666667
Monocyte	PDL-1 on monocyte	Myocardial infarction	15	Inverse variance weighted	−0.03832637	0.96 (0.93–1.00)	0.034768925	21.78377258	0.083167788	0.99610123	0.104666667
Monocyte	CX3CR1 on CD14- CD16 + monocyte	Myocardial infarction	18	Inverse variance weighted	0.031353309	1.03 (1.01–1.06)	0.009775452	13.79361032	0.681653835	0.975205731	0.732
Myeloid cell	CD33dim HLA DR- AC	Myocardial infarction	24	Inverse variance weighted	0.010609557	1.01 (1.00–1.02)	0.036637485	33.0728931	0.079804864	0.135281417	0.100666667
Myeloid cell	Basophil AC	Myocardial infarction	23	Inverse variance weighted	0.011828024	1.01 (1.00–1.02)	0.02244036	32.42877406	0.070374086	0.124820106	0.087
Myeloid cell	CD45 on CD33br HLA DR + CD14-	Myocardial infarction	18	Inverse variance weighted	0.027702335	1.03 (1.00–1.06)	0.048132886	9.244009663	0.932294121	0.447957178	0.936
Myeloid cell	CD45 on CD33- HLA DR-	Myocardial infarction	14	Inverse variance weighted	−0.031921543	0.97 (0.94–1.00)	0.038960777	15.38138857	0.284152125	0.675266364	0.369333333
Myeloid cell	HLA DR on CD33- HLA DR +	Myocardial infarction	15	Inverse variance weighted	0.031016263	1.03 (1.01–1.05)	0.000781554	19.66909402	0.140924077	0.847498067	0.142333333
TBNK	T cell %lymphocyte	Myocardial infarction	17	Inverse variance weighted	−0.043434101	0.96 (0.92–0.99)	0.021342948	19.51073035	0.243069497	0.965558064	0.265
TBNK	T cell %leukocyte	Myocardial infarction	17	Inverse variance weighted	−0.023556297	0.98 (0.95–1.00)	0.04140857	20.97449209	0.17949316	0.470453061	0.309666667
TBNK	DP (CD4 + CD8+) %leukocyte	Myocardial infarction	23	Inverse variance weighted	−0.050316401	0.95 (0.92–0.98)	0.002656635	17.15377494	0.754817823	0.802451369	0.777
TBNK	DN (CD4-CD8-) %leukocyte	Myocardial infarction	21	Inverse variance weighted	0.019673163	1.02 (1.00–1.04)	0.034161547	16.36321187	0.693850955	0.208975141	0.779333333
TBNK	HLA DR + CD8br %T cell	Myocardial infarction	30	Inverse variance weighted	−0.018832243	0.98 (0.97–1.00)	0.017559621	31.7219276	0.332210606	0.761022093	0.386333333
TBNK	HLA DR + CD8br %lymphocyte	Myocardial infarction	31	Inverse variance weighted	−0.028244308	0.97 (0.95–0.99)	0.006364236	41.5993588	0.077417449	0.611822356	0.103333333
TBNK	CD3- lymphocyte AC	Myocardial infarction	18	Inverse variance weighted	−0.058165521	0.94 (0.91–0.98)	0.001723729	26.3284159	0.068681381	0.172558225	0.098
TBNK	NK %lymphocyte	Myocardial infarction	28	Inverse variance weighted	0.022323697	1.02 (1.00–1.04)	0.022479168	25.59226449	0.541307339	0.922552307	0.603
TBNK	CD45 on lymphocyte	Myocardial infarction	24	Inverse variance weighted	0.026997802	1.03 (1.00–1.05)	0.038863788	28.53798069	0.196119132	0.289490433	0.234333333
TBNK	CD45 on NKT	Myocardial infarction	24	Inverse variance weighted	−0.025427282	0.97 (0.96–0.99)	0.014133277	30.70203522	0.130290912	0.272937609	0.161
TBNK	FSC-A on HLA DR + CD4+	Myocardial infarction	18	Inverse variance weighted	0.023837825	1.02 (1.00–1.05)	0.048672315	16.65328862	0.478084755	0.809309064	0.582666667
TBNK	HLA DR on HLA DR + CD8br	Myocardial infarction	21	Inverse variance weighted	0.040393974	1.04 (1.01–1.08)	0.015870883	18.91174871	0.527568948	0.873114218	0.547666667
Treg	CD28- DN (CD4-CD8-) %DN	Myocardial infarction	28	Inverse variance weighted	−0.031457104	0.97 (0.95–0.99)	0.00527594	28.63840613	0.378648049	0.870122562	0.455666667
Treg	CD28+ DN (CD4-CD8-) %DN	Myocardial infarction	28	Inverse variance weighted	0.031457104	1.03 (1.01–1.06)	0.00527594	28.63840613	0.378648049	0.870122562	0.456
Treg	CD28- CD8br %T cell	Myocardial infarction	16	Inverse variance weighted	0.057400377	1.06 (1.02–1.10)	0.005331249	20.09382367	0.16836297	0.091903331	0.161666667
Treg	CD28 on CD28+ CD45RA- CD8br	Myocardial infarction	17	Inverse variance weighted	0.045832708	1.05 (1.01–1.09)	0.022383042	7.224750338	0.968681899	0.546994346	0.975333333
Treg	CD28 on activated Treg	Myocardial infarction	19	Inverse variance weighted	−0.029237451	0.97 (0.95–0.99)	0.007707396	17.96265264	0.458115507	0.339940562	0.492
Treg	CD127 on CD4+	Myocardial infarction	8	Inverse variance weighted	0.058079169	1.06 (1.01–1.11)	0.015812931	11.65957962	0.112324303	0.241866424	0.168666667
Treg	CD25 on resting Treg	Myocardial infarction	19	Inverse variance weighted	−0.027097991	0.97 (0.95–1.00)	0.04141308	15.69907345	0.613539992	0.159459102	0.613666667
Treg	CD39 on granulocyte	Myocardial infarction	27	Inverse variance weighted	0.022770661	1.02 (1.00–1.05)	0.044812238	31.08652414	0.225062706	0.528365583	0.276

#### 3.2.1 B cell

The study revealed that BAFF-R on IgD- CD38br [OR(95% CI):0.96 (0.93–1.00)], CD25 on IgD + CD38br [OR(95% CI): 0.96 (0.93–1.00)], and IgD on IgD + CD38br [OR(95% CI):0.98 (0.96–1.00)] had a protective effect against MI. The risk factors for MI were IgD- CD24- %B cell [OR (95% CI): 1.04 (1.02–1.06)], IgD- CD38br AC [OR (95% CI): 1.02 (1.00–1.04)], CD27 on IgD- CD38br [OR (95% CI): 1.05 (1.00–1.10)], and CD38 on IgD- CD38dim [OR (95% CI): 1.02 (1.00–1.03)] (Table 1).

#### 3.2.2 cDC

The study revealed that four types of DC cells (CD11c + HLA DR++ monocyte % monocyte [OR (95% CI): 1.03 (1.01–1.05)], CD62l- plasmacytoid DC %DC [OR (95% CI): 0.96 (0.93–0.99)], HLA DR on plasmacytoid DC [OR (95% CI): 1.03 (1.01–1.04)], HLA DR on DC [OR (95% CI): 1.03 (1.01–1.04)] were linked to a higher risk of MI. It was also discovered that CD62l on CD62l + myeloid DC [OR (95% CI): 0.96 (0.93–0.99)] was linked to a decreased risk of MI (Table 1).

#### 3.2.3 Maturation stages of T cell

The study revealed that four types of Maturation stages of T cells (EM DN (CD4-CD8-) %DN [OR (95% CI): 1.02 (1.00–1.05)], HVEM on EM CD8br(OR(95% CI):1.03 (1.01–1.05), HVEM on TD CD8br [OR (95% CI):1.02 (1.00–1.04)], CD45RA on naive CD4+ [OR (95% CI): 1.02 (1.00–1.03)] were linked to a higher risk of MI (Table 1).

#### 3.2.4 Monocyte

The study revealed that CD40 on CD14+ CD16 + monocyte [OR (95% CI):0.98 (0.96–0.99)], CD14 on CD14+ CD16 + monocyte [OR (95% CI):0.94 (0.90–0.98)], CD40 on CD14- CD16 + monocyte [OR (95% CI): 0.97 (0.96–0.98)], PDL-1 on monocyte [OR (95% CI): 0.96 (0.93–1.00)] had a protective effect against MI. The risk factors for MI wereCX3CR1 on monocyte [OR (95% CI): 1.03 (1.01–1.06)], CX3CR1 on CD14- CD16 + monocyte [OR (95% CI): 1.03 (1.01–1.06)] (Table 1).

#### 3.2.5 Myeloid cell

The study revealed that four types of DC cells CD33dim HLA DR- AC [OR (95% CI): 1.01 (1.00–1.02)], Basophil AC [OR (95% CI): 1.01 (1.00–1.02)], CD45 on CD33br HLA DR + CD14- (OR (95% CI): 1.03 (1.00–1.06)], HLA DR on CD33- HLA DR+ [OR (95% CI): 1.03 (1.01–1.05)] were linked to a higher risk of MI. It was also discovered that CD45 on CD33- HLA DR- [OR(95% CI):0.97 (0.94–1.00)] was linked to a decreased risk of MI (Table 1).

#### 3.2.6 TBNK

The study revealed that T cell %lymphocyte [OR (95% CI): 0.96 (0.92–0.99)], T cell %leukocyte [OR (95% CI): 0.98 (0.95–1.00)], DP (CD4 + CD8+) %leukocyte [OR (95% CI): 0.95 (0.92–0.98)], HLA DR + CD8br %T cell [OR (95% CI): 0.98 (0.97–1.00)], HLA DR + CD8br %lymphocyte [OR (95% CI): 0.97 (0.95–0.99)], CD3- lymphocyte AC [OR (95% CI): 0.94 (0.91–0.98)], CD45 on NKT [OR (95% CI): 0.97 (0.96–0.99)] had a protective effect against MI. The risk factors for MI were DN (CD4-CD8-) %leukocyte [OR (95% CI): 1.02 (1.00–1.04)], NK %lymphocyte [OR (95% CI): 1.02 (1.00–1.04)], CD45 on lymphocyte [OR (95% CI): 1.03 (1.00–1.05)], FSC-A on HLA DR + CD4+ [OR (95% CI): 1.02 (1.00–1.05)], HLA DR on HLA DR + CD8br [OR (95% CI): 1.04 (1.01–1.08)] (Table 1).

#### 3.2.7 Treg

The study revealed that three types of Tregs cells (CD28- DN (CD4-CD8-) %DN [OR (95% CI): 0.97 (0.95–0.99)], CD28 on activated Treg [OR (95% CI): 0.97 (0.95–0.99], CD25 on resting Treg [OR (95% CI): 0.97 (0.95–1.00)] were linked to a decreased risk of MI, while five types of Tregs cells(CD28+ DN (CD4-CD8-) %DN [OR (95% CI): 1.03 (1.01–1.06)], CD28- CD8br %T cell [OR (95% CI): 1.06 (1.02–1.10)], CD28 on CD28+ CD45RA- CD8br [OR (95% CI): 1.05 (1.01–1.09)], CD127 on CD4+ [OR (95% CI): 1.06 (1.01–1.11)], CD39 on granulocyte [OR (95% CI): 1.02 (1.00–1.05)] were associated with an increased risk of MI (Table 1).

## 4 Discussion

A large proportion of myocardial infarction (MI) is caused by the rupture or erosion of vulnerable atherosclerotic plaques, leading to occlusion of the coronary arteries (39–41). Recent studies have confirmed that atherogenesis and progression of atherosclerosis are associated with inflammation and autoimmunity (42). The pathology of atherosclerosis is characterized by aseptic inflammation. This is mediated by innate and adaptive immune responses (43, 44). Previous studies have shown that innate immunity, represented by monocytes/macrophages, and adaptive immunity, dominated by T/B cells, can accelerate or inhibit atherosclerosis (45). Using MR analysis, we investigated the causal relationship between MI and 731 immune cell traits, based on a significant amount of publicly available genetic data. As far as we know, this study represents the inaugural MR analysis exploring the causal relationship between multiple immunophenotypes and myocardial infarction. In this study, 47 immune cell traits from seven panels were found to be causally associated with MI.

Macrophages are major players in two major cardiovascular diseases, myocardial infarction and atherosclerosis (46). Previous studies have shown significant heterogeneity in macrophage phenotype and function in infarcted myocardium. In the context of atherosclerotic cardiovascular disease (ASCVD), M1 macrophages produce pro-inflammatory cytokines that initiate and maintain inflammation, whereas M2 macrophages produce growth factors and anti-inflammatory cytokines that suppress the immune response (47, 48). However, this is too simplistic to try to categorise. The function of macrophages in the microenvironment of the infarcted heart is currently unknown. It is now believed that macrophages play different roles at different stages of the cardiac inflammatory response (19). Macrophages are recruited to the damaged myocardium and are regulated by the local microenvironment to differentiate into pro-inflammatory and anti-inflammatory macrophages (49–51). Early pro-inflammatory macrophages produce cytokines (e.g., IL-12, IL-23, IL-27, TNF-α) and chemokines (CXCL9, CXCL10, CXCL11) and other chemokines that exert pro-inflammatory effects (52, 53). In contrast, anti-inflammatory macrophages produce mediators such as IL-10, CCL17, and TGF-β during the value-adding and repair processes to exert anti-inflammatory effects, as well as to promote cell proliferation and angiogenesis, and to remove tissue debris (54). At the same time, macrophages crosstalk with cardiomyocytes, fibroblasts and various immune cells to regulate the process of post-infarction myocardial repair (49, 55). Our study found that CX3CR1 on CD14- CD16 + monocyte, CX3CR1 on monocyte were significantly associated with an increased risk of MI, and 4 cell types of monocytes (CD40 on CD14+ CD16 + monocyte, CD14 on CD14+ CD16 + monocyte, CD40 on CD14- CD16 + monocyte, and PDL-1 on monocyte) were associated with a reduced risk of myocardial infarction. These results illustrate the balance between macrophage populations that influence the development of atherosclerosis. This is consistent with previous studies and provides directional targets for future therapy (56–58).

Stimulated by oxidised LDL, TNF-α and hypoxia, DCs enter the vessel wall to take up, process and deliver antigens (59). Our study revealed that four types of DC cells were linked to a higher risk of MI. DCs produce proinflammatory cytokines that activate T and B cells to initiate, modulate and maintain cardiac immunity (60, 61). Coronary plaque rupture is the leading cause of myocardial infarction.DC cells have a potent antigen-presenting effect, activating CD4 + helper cells and CD8 + cytotoxic T cells, killing plaque-resident cells and increasing the risk of plaque rupture (62–64). DC is also involved in shaping the functional activity of natural killer cells, enhancing their cytotoxic potential against endothelial cells and vascular smooth muscle (65). Secondly, DC induces the production of proteases, such as metalloproteinases, which break down the extracellular matrix (62). We found that CD62l on CD62l + myeloid DC was associated with a reduced risk of MI. A study conducted on mice revealed that a depletion of bone marrow dendritic cells (DCs) resulted in a sustained elevation of pro-inflammatory cytokines and a prolonged duration of the inflammatory response following myocardial infarction (66). These results illustrate that different subpopulations of DCs have a division of labour in the development of atherosclerotic sclerosis. It also implies that direct exposure to antigens or soluble cytokines mediates atherosclerosis. Further studies are required to comprehend the pathogenic antigens and DC-derived cytokines linked to cardiovascular disease.

Our research has found that three types of Tregs cells [CD28- DN (CD4-CD8-) %DN, CD28 on activated Treg, CD25 on resting Treg] were linked to a decreased risk of MI. Tregs are major immunoregulatory cells that secrete suppressor cytokines to maintain immune homeostasis. A study has shown that reduced numbers of Tregs and impaired inhibitory function are associated with the progression of atherosclerosis (67). Treg/Th17 ratio, Treg count and Treg function were significantly decreased in acute coronary syndrome (ACS) patients (68). Tregs have also been shown to have a protective effect against atherosclerosis in some studies (69–73). Consistent with our findings, the mRNA sequencing technique showed that Tregs cells play a role in promoting and resisting atherosclerosis (74). Tregs protect against atherosclerosis in different ways. On the one hand, tregs inhibit the progression of atherosclerosis by suppressing the proliferation of T cells that secrete inhibitory cytokines such as IL-10 and TGF-β (72, 75–77). On the other hand, tregs may promote atherosclerotic plaque resolution by activating macrophage cytotoxicity and upregulating pro-soluble lipids (78). In addition, Tregs are involved in regulating lipid metabolism to attenuate the progression of atherosclerosis (79, 80). However our study found that five types of Tregs cells [CD28+ DN (CD4-CD8-) %DN, CD28- CD8br %T cells, CD28+ CD45RA- CD8br on CD28, CD127 on CD4+, CD39 on granulocytes] were associated with an increased risk of myocardial infarction. These results remind us to reconsider the role of Tregs in atherosclerosis, which needs to be further explored by more research, including clinical trials.

B cells are considered a key immune cell type in the regulation of atherosclerosis (81–85). They control the cellular immune response through cell-to-cell contact, antigen presentation, and cytokine production (84). B cell effects mediated by antibodies and cytokines are subpopulation specific. Previous studies have shed light on B cell subpopulations and their functions, demonstrating that distinct subpopulations of B cells have specific effects on atherosclerosis (86, 87). Our study found that 4 B cell types (IgD- CD24- %B cell, IgD- CD38br AC, CD27 on IgD- CD38br, CD38 on IgD- CD38dim) were associated with an increased risk of MI, and 3 B cell types (BAFF-R on IgD- CD38br, CD25 on IgD + CD38br, IgD on IgD + CD38br) reduced the risk of MI.B cells were classified as B1 or B2 cells based on the presence or absence of CD5 expression. Previous studies have shown that B1 cells can improve atherosclerosis by secreting IgM, while B2 cells have proatherogenic effects (84, 88–90). However, B2 cells constitute the majority of B cells and differentiate into follicular B cells (FOB) and marginal zone B cells (MZB). Nus et al. discovered that MZB cells regulate helper T cell responses in hypercholesterolaemic mice and have a protective effect against atherosclerosis (91). B2 cells differentiate into either antibody-producing plasma cells or memory B cells, which secrete antibodies to mediate the humoral immune response. However, studies investigating the correlation between IgG levels and myocardial infarction have produced conflicting results (83). This may be indicative of a specific subtype of IgG. Furthermore, patients with myocardial infarction exhibited elevated plasma IgE levels, indicating a potential proatherosclerotic function for IgE-mediated immune responses (92). Due to the complexity of humoral immunity mediated by B cells that release antigen-specific antibodies, this study provides a new direction for B cell-targeted interventions.

The study is statistically efficient as it is based on the results of a large published GWAS cohort. Two-sample MR analyses and causal inference using multiple MR analyses were used to exclude potential confounders and reverse causation.

However, it is important to note that our study has limitations. However, it must be noted that our study has its limitations. Firstly, Mendelian randomization studies use genetic variation as a natural instrumental variable to infer causality. The presence of pleiotropy means that genetic variation can affect multiple biological pathways or phenotypes, not just the exposure of interest in the study. This can make it difficult to determine whether the effect of a genetic variant is direct or indirect, potentially leading to a misjudgment of the causal relationship between exposure and outcome. To exclude SNPs with pleiotropic effects, we employed the MR-Egger intercept and the MR-PRESSO global test. However, these methods still did not fully control for unknown or unmeasured confounding factors.

Secondly, the relationship between immune responses and cardiovascular health is intricate. In some instances, there may be a linear correlation between immune responses and cardiovascular health; for example, levels of certain inflammatory markers might increase in direct proportion to the rise in cardiovascular risk. The immune system interacts with the cardiovascular system through a variety of cell types and molecular pathways, and these interactions could exhibit different effect patterns at various levels of immune activation. The impact of different types of immune cells on cardiovascular diseases may be independent or interwoven. Moreover, the influence of immune cells on cardiovascular diseases may vary with changes in thresholds, individuals, stages of disease, and other factors. Further research is still needed to address the nonlinear interactions between immunity and cardiovascular diseases.

Additionally, the GWAS data utilized in our study were derived from individuals of European ancestry, hence further investigation is warranted to determine the applicability of our findings to other ethnic groups. The absence of detailed individual information precluded us from conducting refined stratified analyses of the data. Since our study focused solely on specific genetic instruments, it may not fully capture the genetic influence on immune cell activity and the risk of myocardial infarction (MI). With the increasing abundance of genomics data, multi-gene Mendelian randomization studies are demonstrating substantial potential in disease risk prediction, personalized medicine, and the formulation of public health strategies.

## Conclusion

In conclusion, this study provides strong evidence that multiple immune cells have a causal effect on the risk of myocardial infarction. Furthermore, this study highlights the intricate nature of immune cells involved in the development of atherosclerosis and myocardial infarction. This discovery provides a new avenue for the development of therapeutic treatments for myocardial infarction and a new target for drug development.

## Data Availability

The original contributions presented in the study are included in the article/Supplementary Material, further inquiries can be directed to the corresponding author.

## Supplementary material

Supplementary Figure S1 Leave-one-out analysis for the impact of individual SNPs on the association between immune cells and MI risk.

Supplementary Figure S2 The scatter plots and Scatterplots of associations between immune cells and MI risk.
